# The Faroe Islands COVID-19 Recreational Football Study: Player-to-Player Distance, Body-to-Body Contact, Body-to-Ball Contact and Exercise Intensity during Various Types of Football Training for Both Genders and Various Age Groups

**DOI:** 10.1155/2022/6822385

**Published:** 2022-03-17

**Authors:** Magni Mohr, Tórur Sjúrðarson, Eli N. Leifsson, Morten B. Randers, Nikolas Sten Knudsen, Manuel Mounir Demetry Thomasen, Jeppe Panduro, Malte Nejst Larsen, Thomas Bull Andersen, Peter Krustrup

**Affiliations:** ^1^Department of Sports Science and Clinical Biomechanics, SDU Sport and Health Sciences Cluster (SHSC), University of Southern Denmark, Odense, Denmark; ^2^Centre of Health Science, Faculty of Health, University of the Faroe Islands, Tórshavn, Faroe Islands; ^3^School of Sport Sciences, Faculty of Health Sciences, UiT the Arctic University of Norway, Norway; ^4^Section for Sport Science, Department of Public Health, Aarhus University, Aarhus, Denmark; ^5^Shanghai University of Sport, Shanghai, China; ^6^Sport and Health Sciences, University of Exeter, UK; ^7^Danish Institute for Advanced Study (DIAS), University of Southern Denmark, Odense, Denmark

## Abstract

We determined player-to-player distance, body-to-ball contact, and exercise intensity during three training modalities in various football populations. 213 participants were recruited, ranging from 9-year-old boys to young men and 11-year-old girls to middle-aged women. All groups were analysed with video-filming and GPS-based Polar Pro monitors during three types of football training for 20 min, i.e., COVID-19-modified training (CMT) with >2-metre player-to-player distance, small-sided games (SSG), and simulated match-play with normal rules (SMP), in randomised order. Time spent in a danger zone (1.5 m) per-percent-infected-player (DZ PPIP) ranged from 0.015 to 0.279% of playing time. DZ PPIP for SSG was higher (*P* < 0.05) than CMT and SMP. The average number of contacts (within 1.5 m) with a potentially infected player ranged from 12 to 73 contacts/hour. SSG had more (*P* < 0.05) contacts than CMT and SMP, with SMP having a higher (*P* < 0.05) number of contacts than CMT. Time/contact ranged from 0.87 to 3.00 seconds for the groups. No player-to-player and body-to-ball touches were registered for CMT. Total player-to-player contacts were 264% higher (*P* < 0.05) in SSG than SMP, ranging from 80 to 170 and 25 to 56 touches, respectively. In all groups, a greater total distance was covered during SMP compared to CMT (38–114%; *P* < 0.05). All groups performed more high-intensity running (33–54%; *P* < 0.05) and had higher heart rates during SMP compared to CMT. Different types of football training all appear to exert a minor COVID-19 infection risk; however, COVID-19-modified training may be safer than small-sided game training, but also match-play. In contrast, exercise intensity is lower during COVID-19-modified training than match-play.

## 1. Introduction

Over the last fifteen years, a series of studies have demonstrated the immense and clinically significant health-promoting effects of participating in recreational football across a person's lifespan [1–3]. Indeed, solid meta-analysis-level evidence shows that regular participation in team sports induces broad-spectrum health effects in sedentary people ranging between 18 and 80 years [2]. Moreover, team sports training can be included in the treatment of several chronic diseases, such as numerous cardiovascular diseases [3, 4], type 2 diabetes [5], some cancer types [6], and osteopenia [7]. Finally, strong scientific evidence documents the beneficial effects of football participation on children in terms of physical fitness and health profile [8, 9], social well-being, and mental health [10, 11].

The physical inactivity pandemic is a global public health challenge. One-third of the world population is failing to meet the minimum recommendations for physical activity [12], which causes approximately 3.2 million deaths every year [13]. Moreover, physical inactivity is increasing [14]; for example, 80% of children between 13 and 15 years of age are physically inactive. The new COVID-19 pandemic, which resulted in home confinement and restricted participation in sports imposed by the authorities, has led to major changes in the pattern of physical activity [13, 15]. For example, a more than 30% decrease in physical activity has been reported with large ranges between countries due to different national restriction initiatives [16]. Moreover, large increases in sitting and screen time among both children and adults have been observed, indicating a marked rise in sedentary behaviour [17, 18].

Football is the most popular sport, with more than 265 million footballers around the globe, and the vast majority involved in amateur and recreational football [19]. As stated above, football plays a central role in public health promotion. However, the game has been considered as a contact sport, with frequent and close contacts between players during training and games and is therefore considered to put players at high risk of disease transmission [20]. Authorities may therefore be reluctant to allow sports such as football. Yet the consideration that football is a contact sport, with frequent and close contact, is built not upon scientific evidence, but upon an assumption. As such, it is important to investigate safe methods for team sport participation for various population groups.

Recently, tracking data has been shown to be a viable method for assessing exposure to interpersonal contact in sports [21] and studies have calculated an exposure score to COVID-19 transmission during football match-play based on the tracking of players using a high temporal-resolution (25 Hz) tracking system measuring a defined “danger zone” (DZ) for respiratory infections [21, 22]. These studies found that the mean or median exposure time was 44–87 seconds during professional matches, which results in a low COVID-19 infection risk during the actual game [21, 22]. Moreover, we recently demonstrated that limited time is spent within a DZ during small-sided game training and that these contacts are brief [23]. Thus, recreational football may be better defined as a sport with sporadic brief contacts. However, little is known about the exposure time to potential COVID-19 infection during normal recreational football training and “COVID-19-modified training” (drills adjusted to maintain distance between the players) at different levels in different population groups. The aim of this study is therefore to determine exposure time and contact during normal football training, COVID-19 training, and match-play for children and adult competitive and recreational players. The Faroe Islands and South Korea were the first countries in the world to allow football training and match-play again after the COVID-19 lockdown. Therefore, this study was performed on the Faroe Islands in June 2020 immediately after the lockdown under the first wave of COVID-19.

## 2. Materials and Methods

### 2.1. Participants

Three U9 male teams (U9B; *n* = 50), three U11 female teams (U11G; *n* = 43), three U14 male teams (U14B; *n* = 51), two competitive young male teams (YM; *n* = 43), and two recreational female teams (RF; *n* = 26) participated (in total 213). All participants were informed about the potential risks and benefits of their participation in the data collection. The study was conducted in accordance with the Declaration of Helsinki (1964) and was approved by the local ethics committee. All players (or their parents if below 18 years) gave their written informed consent to participate.

### 2.2. Experimental Design

There were two experimental days per team/player. *Day 1* started with a 15 min standardised warm-up consisting of jogging, strength exercises, dynamic stretching, and agility runs followed by 20 min comprising each of three training types performed in a randomised order. The subsequent training types were (1) COVID-19-modified training, (2) traditional training (without any body contact or distance restrictions), and (3) match-play.

*COVID-19-modified training* (CMT) consisted of technical drills such as passing and dribbling drills where players were instructed to maintain a minimum 2 m distance and pitch size was regulated accordingly.

*Traditional training* consisted of small-sided games (SSG) divided into 10 min possession drills with rules and without goals, followed by 10 min of 3v3-5v5 depending on the total number of participants, and the pitch size was adjusted to 32 m^2^ per player.

*Simulated match-play* (SMP) consisted of a 20-minute football match with normal rules. Pitch size was regulated according to the age groups.

Participant wore Polar Pro unite with 10 Hz GPS and heart rate monitors (Polar Electro Oy, Kempele, Finland). The precision and reproducibility of high temporal resolution GPS systems have been described previously [24]. All of the sessions were filmed for subsequent player-to-player and body-to-ball contact analysis and technical analysis.

### 2.3. Danger Zone Calculation

X- and Y-coordinates were retrieved from the tracking data, and the data were filtered using a Butterworth fifth-order low-pass filter with a cut-off frequency of 0.08 Hz using a build-in MatLab function (The MathWorks, Inc, New York, USA). To evaluate the risk of being infected, a danger zone (DZ) was constructed as a circle with a radius of 1.5 m around each players' position. In addition to the circular zone, a tail followed each player as an area the player was positioned in up to 6 s ago. The danger value of this tail declines exponentially with a half-life of 2 s as gravity pulls the droplets towards the ground [25, 26]. Thus, being within 1.5 m of another player returns a danger score of 1, whereas being in the area where the other player was 2 and 4 s ago equates to a danger score of 0.5 and 0.25, respectively. If a player is within multiple zones at the same time, the score is determined as the maximum score of the zones. Accordingly, the maximum danger score at any time and position is 1. An exposure score is calculated based on the sum of all danger scores divided by the sample frequency (10 Hz), which is then translated to how much time a player spent in a DZ throughout the game. Calculations were performed with one infected player in each game and repeated until all participants had acted as the infected player as previously described [22, 23]. Moreover, the number of contacts was evaluated as the number of times a player entered into a DZ, and the duration of each entry was noted.

### 2.4. Player-to-Player Touches and Body Contact Assessment

Player-to-player touches and body-to-ball touches were evaluated using video analysis. The player-to-player touches were categorised into nine different subgroups based on different types of touches: front-front, front-side, front-back, side-side, back-side, back-back, hand-shoulder, hand-thigh, and tackle-foot. Likewise, body-to-ball touches were categorised into four different subgroups: headers, ball-hand, ball-thigh, and ball-chest. Every time a player touched another player or touched the ball with other body parts than the foot, the video was stopped, and the type noted.

### 2.5. Activity Pattern

Positional data and heart rate were also collected using Polar Team Pro GPS-unit sampling at 10 Hz on all participants during the warm-up and the three training methods. Total distance covered was measured. High-intensity running was categorised as the distance covered at 12–16 km/h for children, 13–16 km/h in women playing for recreation, and 13–20 km/h for young men. Sprint distance was categorised as the distance covered at >16 km/h for children and women playing for recreation and at >20 km/h for young men. Accelerations counts are divided into two categories for children: low (1.50–2.30 m/s^2^) and high (>2.30 m/s^2^). Acceleration counts are divided into three categories for adults: low (1.50–2.14 m/s^2^), medium (2.14–2.78 m/s^2^), and high (>2.78 m/s^2^).

### 2.6. Statistical Analysis

Video analysis data are presented as mean ± SD unless stated otherwise. Differences between the three types of gameplay were evaluated using one-way analysis of variance (ANOVA). When a significant difference was detected, post hoc testing was performed using Bonferroni correction. GPS and HR data are presented by means [±95% confidence intervals]. Data are presented as percentage time in DZ and s h^−1^ to compare SSG of different durations. Moreover, to compare SSG with different number of participants, the data are presented as per percentage infected player (PPIP).

### 2.7. Patient and Public Involvement

Patients and/or the public were not involved in the design, or conduct, or reporting, or dissemination plans of this research.

## 3. Results

### 3.1. Danger Zone Assessments

**Figure 1:**
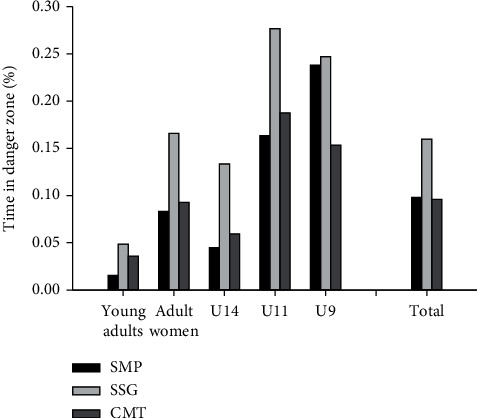
Percentage time spent in danger zone per percent infected player during simulated match-play (SMP), small-sided games (SSG), and corona-modified training, (CMT) U9 boys (*n* = 50), U11 girls (*n* = 43), U14 boys (*n* = 51), young adult men (*n* = 43), and adult women (*n* = 26), as well as for the total study population (*n* = 213).

Percentage time spent in DZ PPIP for all participating teams across the three activities ranged from 0.015 [0.014; 0.017] to 0.279 [0.243; 0.306] %. For the entire group, time in DZ PPIP for SSG was higher (*P* < 0.05) than CMT and SMP, with no difference between CMT and SMP (Figure 1). However, when comparing time in DZ PPIP during the activities for each level of play, YM and U14B had a higher (*P* < 0.05) time in DZ PPIP in SSG than CMT and SMP, with higher (*P* < 0.05) time in DZ PPIP in CMT compared to SMP. RF and U11G had a higher (*P* < 0.05) time in DZ PPIP during SSG than both CMT and SMP, with no difference between CMT and SMP. U9B had a lower (*P* < 0.05) time in DZ PPIP in CMT than both SSG and SMP, with no difference between SSG and SMP (Figure 1).

**Figure 2:**
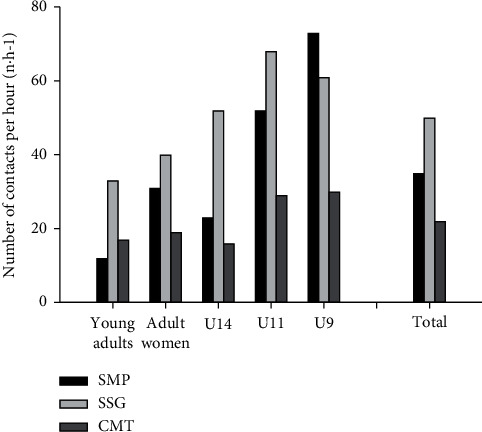
Number of contacts per hour during simulated match-play (SMP), small-sided games (SSG), and corona-modified training (CMT), U9 boys (*n* = 50), U11 girls (*n* = 43), U14 boys (*n* = 51), young adult men (*n* = 43), and adult women (*n* = 26), as well as for the total study population (*n* = 213).

**Figure 3:**
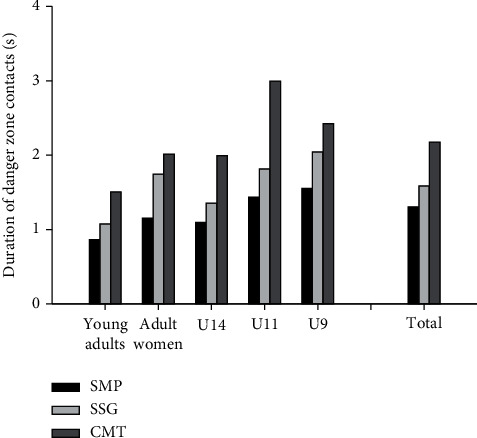
Duration of contacts in danger zone during simulated match-play (SMP), small-sided games (SSG), and corona-modified training (CMT), U9 boys (*n* = 50), U11 girls (*n* = 43), U14 boys (*n* = 51), young adult men (*n* = 43), and adult women (*n* = 26), as well as for the total study population (*n* = 213).

The average number of entries into the DZ of the infected player, assuming that one participant was infected, ranged from 12.0 [10.8; 13.1] to 73.0 [68.8; 76.2] entries per hour. For the entire group, a higher (*P* < 0.05) number of entries into a DZ per hour was observed in SSG than CMT and SMP, with SMP having higher (*P* < 0.05) numbers than CMT. This was observed across all playing levels, except for U9B, where SMP showed a higher (*P* < 0.05) number of entries into a DZ per hour than both SSG and CMT, with SSG having higher (*P* < 0.05) numbers than CMT (Figure 2). Time per entry into a DZ ranged from 0.87 [0.84; 0.90] to 3.00 [2.88; 3.12] seconds across teams and activities. For the entire group and for all teams, CMT had a longer (*P* < 0.05) time per entry into a DZ than SSG and SMP, with SSG having a longer (*P* < 0.05) time per entry into a DZ than SMP (Figure 3).

### 3.2. Body Contacts and Body-to-Ball Touches

No player-to-player and body-to-ball touches was registered for the CMT intervention (Table 1). Total player-to-player touches were 264% higher (*P* < 0.05) in SSG than in SMP, ranging from 80 to 170 and 25 to 56 touches, respectively (Table 2). In SMP, the fewest player-to-player touches were observed in YM (25 touches) and the highest number of touches in U11G (56 touches). In SSG, total player-to-player touches ranged from 80 (RF) to 170 (YM) touches. Average front-to-front touches as a percentage of total touches were not significantly different between game type (9% and 10% for SMP and SSG, respectively) ranging from 0 to 18% in SMP and 7 to 17% in SSG. The number of player-to-player touches per player per hour ranged from 3.3 to 12.1 for SMP and 18.1 to 22.2 for SSG, averaging 8.3 and 21.3 touches for SMP and SSG, respectively.

Body-to-ball touches per player per hour were not significantly different between game type, with an average of 4.0 and 3.8 body-to-ball touches per player per hour for SMP (range: 0.3–6.5) and SSG (1.4–8.9), respectively. Likewise, the various activities did not differ in the number of headers and ball-to-hand touches. The player-to-player and body-to-ball contacts of different activities and teams are presented in Table 2.

### 3.3. Exercise Intensity and Heart Rate Loading

In all groups, a greater total distance (range: 38–114%; *P* < 0.05) was covered during SMP compared to CMT (Table 3). In U9B, U14G, and RF, a greater distance (24–87%; *P* < 0.05) was covered during SSG compared to CMT. Finally, U11G, U14G, YM, and RF covered more ground (14–67%; *P* < 0.05) during SMP than SSG (Table 3). All groups also covered a greater high-intensity running distance (33–54%; *P* < 0.05) during SMP compared to CMT, whereas only U9B ran 74% longer at high intensity (*P* < 0.05) during SSG compared to CMT (Table 3). All groups performed 2–4 times more (*P* < 0.05) high-intensity running during SMP compared to SSG, whereas a higher (*P* < 0.05) sprint distance was observed in SMP than CMT (77–100%) and SSG (75–99%). Several differences were also observed in the number of accelerations between interventions (Table 3). 8–11% higher (*P* < 0.05) HR was observed in U9B, YM, and RF during SMP than CMT, whereas only YM and RF experienced higher heart rates (6 and 10%, respectively; Table 3) during SSG compared to CMT.

## 4. Discussion

This study is the first to examine player-to-player distance, body-to-body, and ball contacts and exercise intensity during various types of football training for both genders and different age groups. The principal findings were that various types of football training across different populations can be considered a relatively safe activity in relation to the risk of COVID-19 infection. Nevertheless, the infection risk appears to be lower when football training is organised as COVID-19-modified training, especially compared to small-sided game training, but also during match-play. COVID-19-modified training, however, elicited lower exercise intensity and physiological loading compared to small-sided game training and match-play.

These findings, which suggest that football training is a safe activity in relation to the transmission risk of COVID-19, are supported by recent analyses of recreational football [23] and elite football match-play [22]. In our study, relative time spent in the danger zone per percent infected player ranged from only 0.02 to 0.28% of total playing time, depending on training modality and football population. Moreover, the average frequency of entries into the danger zone of an infected player, assuming one participant is infected, ranged from 12 to 73 entries per hour, while time per entry into the danger zone was 1–3 seconds on average across teams and activities. These results are reinforced by Randers et al. [23], where time spent in the danger zone during 1-hour recreational football sessions was 4–8 seconds per percent infected players, corresponding to 35–115 seconds per hour if one player was infected. The study in question also reported that around 25–85 contacts occur per hour, with an average contact time of only 1.0–1.5 seconds. Thus, in general, the frequency of entries and time spent in the danger zone, during which COVID-19 transmission can occur, is low during football training for a broad range of populations.

Our study also compared three different training methods in relation to the markers of estimated COVID-19 transmission risk. The GPS recording demonstrated that in general, players stayed longer and had more entries into the danger zone during small-sided game technical training drills compared to the simulated match-play and COVID-19-modified training. This is further supported by no player-to-player and body-to-ball touches during COVID-19-modified training and 2.5 times higher touches during small-sided games compared to match-play (80–170 vs 25–56). Collectively, COVID-19-modified training and match-play appear to be safe training protocols for football players ranging from young children to adults, while the risk may be slightly higher during traditional small-sided technical drills. There were surprisingly few differences between the COVID-19-modified training, which was mainly aimed at maintaining social distancing, and simulated match-play. This confirms previously mentioned findings that football match-play is a relatively safe activity in relation to COVID-19 infection risk [22, 23].

In accordance with a series of previous studies examining the intensity and training-induced fitness and health effects of recreational football [1–4], only minor differences were observed between the different football populations, with high average and peak heart rate loading and multiple high-intensity runs and football-specific intense actions, such as direction changes, dribbles, and shots. The heart rate data showed a minor difference between average heart rate and time spent over 80% and 90% of maximal heart rate during small-sided technical drills and simulated match-play, which supports the findings by others investigating different populations [27]. Somewhat lower values were observed in COVID-19-modified training. In contrast to the other participant groups, middle-aged women showed the most time spent above 80% of maximal heart rate during COVID-19-modified training, which may be explained by the fact that this group is less fit and has less experience of conventional football training than the other groups. In general, therefore, cardiovascular loading appears to be lower during COVID-19-modified training. Moreover, total distance covered was lower during COVID-19-modified training compared to small-sided games and match-play for most participant groups, while high-intensity running, sprinting, and intense accelerations appeared highest during match-play and lowest in COVID-19-modified training. Thus, match-play provides a markedly higher training load for a wide range of football populations when compared to COVID-19-modified training.

Our findings emphasise the potential of football as a versatile and effective type of training that can be used to provide broad-spectrum fitness, well-being, and health effects for a variety of population groups. The data support other observations in children [28], sedentary adults [1–3], elderly [29, 30], and various patient groups with hypertension, type 2 diabetes, and cancer [31–33]. In most regions, the COVID-19 pandemic has reduced physical activity among the general public [16] and simultaneously increased sedentary and screen time in children and adults [17, 34]. Football training can thus be utilised to boost public health during periods with increased sedentary behaviour, such as long intervals with COVID-19-induced restrictions in social gatherings. The newly developed COVID-19-modified training may be the safest training method, but it may not provide the same potent training stimulus as more conventional training methods. However, COVID-19-modified training can be prescribed for patient groups who have to take extra precautions against COVID-19. Moreover, match-play seems to be both safe and offer optimal cardiovascular, metabolic, and musculoskeletal loading. As a worst-case scenario, as an alternative to completed lockdown, health authorities may, in conjunction with sporting organisations and clubs, seek to regulate the type of football training conducted by adopting COVID-19-modified training, taking into account the infection risk at the given time.

In conclusion, different types of football training appear to entail a minor COVID-19 infection risk. The COVID-19-modified training can be used to minimize the risk even further has an even lower risk although it should be noted that the exercise intensity is lower during COVID-19-modified training than match-play. Taken together, the present study suggests that recreational football may be recommended as a low-transmission outdoor sporting activity with sporadic contact and multiple training effects for a variety of male and female participant groups.

## Figures and Tables

**Table 1:** Types of touches during simulated match-play (SMP), small-sided games (SSG), and corona-modified training (CMT) as average of all groups (U9 boys (*n* = 50), U11 girls (*n* = 43), U14 boys (*n* = 51), young adult men (*n* = 43), and adult women (*n* = 26)), as well as average of all games.

Age group	Average all participant groups			
Game type	CMT	SSG	SMP	Total average
Players (*n*)	16	16	16	16
Time (min)	20	20	20	60
Total player-to-player touches	0	116	44	160
Front-front touches	0	11	4	15
Front-to-front % of total	0%	9%	10%	9%
Player-to-player touches/player	0.0	7.1	2.8	9.8
Touches/player/hour	0.0	21.3	8.3	29.5
Front-front touches/player/hour	0.0	2.0	0.8	2.8
Body-to-ball touches	0	21	22	42
Headers	0	7	5	12
Ball-to-hand	0	11	16	27
Body-to-ball touches/player	0.0	1.3	1.3	2.6
Body-to-ball touches/player/hour	0.0	3.8	4.0	7.9
Headers/player/hour	0.0	1.2	0.9	2.2
Ball-to-hand/player/hour	0.0	2.0	2.9	5.0
Player-to-player touches/player/minute	0.00	0.35	0.14	0.16
Front-front touches/player/minute	0.00	0.03	0.01	0.02

**Table 2:** Types of touches during simulated match-play (SMP) and small-sided games (SSG) for U9 boys (*n* = 50), U11 girls (*n* = 43), U14 boys (*n* = 51), young men (*n* = 43), and recreational females (*n* = 26).

Age group	U9 B	U9 B	U11 G	U11 G	U14 B	U14 B	YM	YM	RF	RF
Game type	SSG	SMP	SSG	SMP	SSG	SMP	SSG	SMP	SSG	SMP
Players (*n*)	17	17	15	15	15	15	23	23	12	12
Time (min)	20	20	20	20	20	20	20	20	20	20
Total player-to-player touches	98	55	105	56	96	45	170	25	80	49
Front-front touches	7	10	9	5	7	2	15	0	14	6
Front-to-front % total	7%	18%	9%	10%	7%	6%	9%	0%	17%	12%
Player-to-player touches/player	6.0	3.3	7.0	3.7	6.4	3.0	7.4	1.1	6.7	4.0
Touches/player/hour	18.1	10.0	20.9	11.2	19.1	8.9	22.2	3.3	20.1	12.1
Front-front touches/player/hour	1.2	1.8	1.9	1.1	1.4	0.5	2.0	0.0	3.6	1.2
Body-to-ball touches	24	30	43	31	9	22	15	19	15	1
Headers	8	5	1	1	4	9	10	8	10	0
Ball-to-hand	15	25	40	29	3	10	0	11	0	1
Body-to-ball touches/player	1.5	1.9	3.0	2.2	0.5	1.1	0.7	0.8	1.3	0.1
Body-to-ball touches/player/hour	4.5	5.6	8.9	6.5	1.4	3.4	2.0	2.5	3.8	0.3
Headers/player/hour	1.5	1.0	0.1	0.1	0.6	1.3	1.3	1.0	2.5	0.0
Ball-to-hand/player/hour	2.8	4.6	8.3	6.1	0.5	1.6	0.0	1.4	0.0	0.3
Player-to-player touches/player/minute	0.30	0.17	0.36	0.19	0.24	0.12	0.37	0.05	0.33	0.20
Front-front touches/player/minute	0.02	0.03	0.03	0.02	0.02	0.02	0.03	0.00	0.06	0.02

**Table 3:** Game activities and heart rate loading during the three training protocols in all groups. High-intensity running categorised as distance covered between 12 and16 km/h for children, between 13 and 16 km/h for recreational females, and between 13 and 20 km/h for young men. Sprint distance categorised as the distance covered above >16 km/h for children and recreational females and as distance covered above >20 km/h for young men. Acceleration counts are divided into two categories for children, low (1.50–2.30 m/s^2^) and high (2.30 m/s^2^). Acceleration counts are divided into three categories for adults, low (1.50–2.14 m/s^2^), medium (2.14–2.78 m/s^2^), and high (>2.78 m/s^2^).

	U9 B			U11 G			U14 B			Young men			Women (recreational)		
	CMT (*n* = 43)	SSG (*n* = 44)	SMP (*n* = 43)	CMT (*n* = 41)	SSG (*n* = 41)	SMP (*n* = 41)	CMT (*n* = 49)	SSG (*n* = 49)	SMP (*n* = 50)	CMT (*n* = 42)	SSG (*n* = 42)	SMP (*n* = 41)	CMT (*n* = 25)	SSG (*n* = 26)	SMP (*n* = 49)
Total distance (m)	579 ± 290^#^	844 ± 160^Æ^	801 ± 273^Æ^	401 ± 47	751 ± 123^Æ^	857 ± 253^Æ,Ø^	1015 ± 129^∗^^,#,^^	1114 ± 394^∗^^,#,^^	1856 ± 494^Æ,Ø,^^∗^^,#,^^	1515 ± 263^∗^^,#,&,^^	1558 ± 119^∗^^,#,&,^^	2272 ± 525^Æ,Ø,^^∗^^,#,&,^^	600 ± 68^#^	745 ± 157^Æ^	1027 ± 187^Æ,Ø^
High intensity running (m)	10 ± 16	35 ± 24^Æ^	76 ± 62^Æ,Ø^	4 ± 7	16 ± 13	57 ± 48^Æ,Ø^	60 ± 57^∗^^,#,^^	54 ± 42^#,^^	282 ± 134^Æ,Ø,^^∗^^,#,^^	154 ± 87^∗^^,#,&,^^	121 ± 53^∗^^,#,&,^^	501 ± 235^Æ,Ø,^^∗^^,#,&,^^	7 ± 7	12 ± 13	41 ± 21^Æ,Ø^
Sprint distance (m)	4 ± 13	3 ± 8	18 ± 23^Æ,Ø^	0 ± 2	1 ± 2	9 ± 13^Æ,Ø^	8 ± 16^#,¤^	6 ± 7^#^	174 ± 106^Æ,Ø,^^∗^^,#,¤,^^	1 ± 2	3 ± 4	132 ± 81^Æ,Ø,^^∗^^,#,^^	1 ± 4	4 ± 7	16 ± 15^Æ,Ø^
Accel. Count (low)	4 ± 4	15 ± 7^Æ,^^	13 ± 8^Æ^	2 ± 2	11 ± 6^Æ^	12 ± 8^Æ^	13 ± 9^∗^^,#^	22 ± 13^Æ,^^∗^^,#,^^	26 ± 11^Æ,^^∗^^,#,^^	40 ± 12^∗^^,#,&,^^	38 ± 10^∗^^,#,&,^^	35 ± 11^∗^^,#,&,^^	9 ± 6^#^	9 ± 5	11 ± 6
Accel. Count (medium)										10 ± 7^^^	9 ± 4^^^	11 ± 5^^^	0 ± 1	2 ± 2^Æ^	2 ± 2^Æ^
Accel. Count (high)	0 ± 0	1 ± 1^Æ,^^	1 ± 1^Æ^	0 ± 0	1 ± 1^Æ^	1 ± 1^Æ^	1 ± 2^∗^^,#,^^	2 ± 2^#,^^	3 ± 3^Æ,Ø,^^∗^^,#,¤,^^	1 ± 2^∗^^,#,^^	1 ± 1^^^	2 ± 2^Æ,^^∗^^,#,^^	0 ± 0	0 ± 1	0 ± 0
Mean HR	146 ± 13	153 ± 14	157 ± 18^Æ^	156 ± 13^∗^^,&,¤,^^	162 ± 14^^^	160 ± 18	148 ± 10	163 ± 13^∗^^,^^	170 ± 15^∗^^,^^	146 ± 14	161 ± 11^Æ,^^	162 ± 15^Æ^	141 ± 13	150 ± 14^Æ^	152 ± 14^Æ^
Max HR	177 ± 17^^^	187 ± 16^Æ,^^	185 ± 16^^^	181 ± 14^¤,^^	187 ± 15^^^	186 ± 16^¤,^^	178 ± 11^¤,^^	185 ± 12^^^	193 ± 12^¤,^^	168 ± 17	183 ± 12^Æ,^^	183 ± 14^Æ^	161 ± 14	167 ± 13	173 ± 17^Æ^
% > 80 HRmax	10	19	22	22	33	28	14	35	36	26	39	38	38	30	28
% > 90 HRmax	1	3	6	3	7	10	2	13	26	3	19	24	18	40	42

Differences inside group between activities: ^Æ^significantly larger than CMT. ^Ø^significantly larger than SSG. ^Å^significantly larger than SSG. Differences between groups: ^∗^significantly larger than U9. ^#^significantly larger than U11. ^&^significantly larger than U14. ^¤^significantly larger than young men. ^^^significantly larger than recreational females.

## Data Availability

Readers can get access the data supporting the conclusions of the study by contacting the corresponding author.
